# Attitudes and Satisfaction toward the Taken Procedures to Tackle COVID-19 Pandemic in Palestine

**DOI:** 10.4314/ejhs.v32i1.4

**Published:** 2022-01

**Authors:** Samer Abuzerr, Kate Zinszer, Amira Shaheen, Abdel Hamid El Bilbeisi, Alshaarawi Salem, Ali Aldirawi, Osama Jabr Emad, Ayman Al Haj Daoud, Rima Naser, Kamal Eldeirawi

**Affiliations:** 1 Visiting Scholar, Department of Social and Preventive Medicine, University of Montreal, Montréal, Canada; 2 Quality Improvement and Infection Control Unit, Ministry of Health, Gaza, Palestine; 3 School of Public Health, Department of Social and Preventive Medicine, University of Montreal, Canada; 4 Public Health Department, Faculty of Medicine and Health Sciences, An-Najah National University, Palestine; 5 Department of Nutrition, School of Medicine and Health Sciences, University of Palestine, Gaza Strip, Palestine; 6 Optometry and vision sciences, University of Minho, Portugal; 7 Pediatric Intensive Care Unit, Al-Shifa Hospital, Ministry of Health, Gaza, Palestine; 8 Mental Health General Directorate, Ministry of Health, Gaza, Palestine; 9 Palestine Academy for Science and Technology (PALAST), West Bank, Palestine; 10 Ph.D. Candidate, Universidade do Minho; 11 Department of Population Health Nursing Science, UIC College of Nursing (M/C 802), Chicago, USA

**Keywords:** COVID-19, Palestine, Attitudes, Procedures, Satisfaction

## Abstract

**Background:**

Since the beginning of the COVID-19 pandemic, there have been differences in the mitigation strategies implemented by governments worldwide. In addition, people's acceptance and adherence to these strategies, such as avoiding large gatherings and shelter in place, varied. The current study aims to assess the attitude and satisfaction with the procedures to tackle COVID-19 in Palestine.

**Methods:**

This cross-sectional descriptive study was conducted in the Palestinian territories, including, Gaza Strip, West Bank, and East Jerusalem, between April 29, 2020, and June 5, 2020, using a validated online questionnaire. The questionnaire included three sections: socio-demographic characteristics, attitude towards the measures and behaviors to avoid COVID-19 infection and its consequences, and level of people satisfaction with the response of the community and local authorities to combat the COVID-19 pandemic. A convenience sampling method was used to select participants. Statistical analysis was performed using SPSS version 26.

**Results:**

A total of 570 adults aged ≥18 years (56.3% males and 43.7% females) were included in the study. The mean positive attitude score (average % agree or strongly agree) was 94.22%; 95.24%, 95.18%, and 92.18% in the Gaza Strip, West Bank, and East Jerusalem, respectively. While, the mean satisfaction score was 44.26%, distributed as 47.16%, 46.1%, and 39.22% in the Gaza Strip, West Bank, and East Jerusalem, respectively. Additionally, there were statistically significant variations by most attitude and satisfaction variables across the governorates included in the study (p < 0.05). The current study demonstrated high levels of positive attitude but suboptimal level of satisfaction toward the taken procedures to tackle COVID-19 in Palestine.

**Conclusions:**

Varied implementation strategies to improve the levels of satisfaction toward the approaches to combat the COVID-19 pandemic are recommended.

## Introduction

The COVID-19 pandemic has had a detrimental impact on people's mental and physical well-being. The pandemic has been linked with symptoms of posttraumatic stress disorder, loneliness, depression, anxiety, fatigue and insomnia, resulting from self-isolation, quarantine, and exposure to social media with negative news. These have become a threat to the physical and mental health of people (1,2). In Palestine, the health care system is highly fragmented, with a severe shortage of resources that will negatively affect efforts to tackle the COVID-19 pandemic (3,4). The Palestinian health care system consists of four health care service providers: the Palestinian Ministry of Health, which is the leading and primary provider; the United Nations Relief and Works Agency for Palestine Refugees in the Near East (UNRWA); non-governmental organizations; and the private sector (5). Some of the issues facing this system include its decreased ability to rapidly develop evidence-based policies and share/coordinate information in addition to its inability to utilize modern technologies such as electronic medical records. These components are essential for the development and implementation of contingency plans and measures to confront epidemics, especially given the limited resources and decreased health supplies currently experienced in Palestinian hospitals (6–8).

Since the beginning of the COVID-19 pandemic, differences in the mitigation strategies implemented by governments around the globe have been noted. Palestine has implemented several rapid and strict procedures and measures to control and reduce the spread of COVID-19. These measures included social distancing, wearing face masks, closing non-essential businesses, travel bans, suspending religious and prayer services at places of worship, quarantine for patients and contacts, the COVID-19 pandemic has had a detrimental impact on people's mental and physical well-being. The pandemic has been linked with symptoms of posttraumatic stress disorder, loneliness, depression, anxiety, fatigue and insomnia, resulting from self-isolation, quarantine, and exposure to social media with negative news. These have become a threat to the physical and mental health of people (1, 2). In Palestine, the health care system is highly fragmented, with a severe shortage of resources that will negatively affect efforts to tackle the COVID-19 pandemic (3). The Palestinian health care system consists of four health care service providers: the Palestinian Ministry of Health, which is the leading and primary provider; the United Nations Relief and Works Agency for Palestine Refugees in the Near East (UNRWA); non-governmental organizations; and the private sector (5). Some of the issues facing this system include its decreased ability to rapidly develop evidence-based policies and share/coordinate information in addition to its inability to utilize modern technologies such as electronic medical records. These components are essential for the development and implementation of contingency plans and measures to confront epidemics, especially given the limited resources and decreased health supplies currently experienced in Palestinian hospitals (6–8).

Since the beginning of the COVID-19 pandemic, differences in the mitigation strategies implemented by governments around the globe have been noted. Palestine has implemented several rapid and strict procedures and measures to control and reduce the spread of COVID-19. These measures included social distancing, wearing face masks, closing non-essential businesses, travel bans, suspending religious and prayer services at places of worship, quarantine for patients and contacts, and curfews as well as limiting movement between different geographic areas. These procedures were necessary measures and precautions to prevent a sudden spread in cases (9).

Scientific studies indicated that quarantine, travel restrictions and wearing face masks were the most effective preventive measures and procedures to control the spread of infection (10, 11). Findings of a recent online community-based cross-sectional survey of Palestinians conducted by Abuzerr et al. (2021) demonstrate that over 70% of respondents indicated that the COVID-19 pandemic had had a heavy burden on their families, suggesting a need for more attention to the mental/emotional as well as physical health needs of Palestinians (12). It is essential to understand people's attitudes toward these strategies and their impact (13). In addition, the promotion of a positive social environment may depend, in part, on how people perceive these public health policies and systems (14). Negative attitudes toward infectious illnesses and decreased health knowledge may negatively influence efforts to control the spread of infection (15).

Therefore, it is crucial to understand the attitudes of people toward and their satisfaction with the COVID-19 mitigation measures and policies in order to increase the effectiveness of these preventive measures and procedures. People's satisfaction with and confidence in governmental efforts are two critical conditions for accomplishing practical application and adherence to future standards. On the other hand, reduced people satisfaction with or confidence in their governments and their policies may have a negative impact on and deter the fight against the COVID-19 pandemic (16). Therefore, this study aimed to understand and assess the attitudes and satisfaction toward the procedures taken to tackle COVID-19 in Palestine. This information may improve the response to the COVID-19 pandemic as well as other future pandemics and catastrophes.

## Methods

**Study population**: This cross-sectional descriptive study was conducted online in the Palestinian territories, including Gaza Strip, West Bank, and East Jerusalem, between April 29, 2020, and June 5, 2020.

**Data collection**: A comprehensive questionnaire in Arabic was distributed via social media, particularly in the Gaza Strip, West Bank, and East Jerusalem. Participants were invited to answer a structured online survey developed by the University of Coimbra, Portugal, via the Health Geography Research Team at the Centre of Studies in Geography and Spatial Planning (CEGOT) (17). The study survey included a checklist of socio-demographic variables as well as population characteristics, questions on people's attitude about the measures and behaviors to avoid infection with COVID-19 and its consequences, and questions to assess the level of the participants' satisfaction with the response of the community and local authorities to combat COVID-19 pandemic. Four-point Likert-type scale was used to get responses regarding participant's attitude whereas five-point Likert-type scale was used to get responses regarding participants satisfaction.

Participants who answered by agree/strongly agree to a series of items were considered to have positive attitude and participants' responses were averaged to calculate the average percent with positive attitude. Similarly, participants who answered by satisfied/strongly satisfied to a series of items were considered to be satisfied and participants' responses were averaged to calculate the average percent of participants who were satisfied.

The survey was distributed through multiple groups and social media pages to collect the maximum number of participants. Potential participants interested in the survey were asked to click on the URL or link for the survey.

**Eligibility criteria**: Palestinian adults aged ≥18 years old residing in the Gaza Strip, West Bank, and East Jerusalem were invited to complete the online survey. The participants who answered the survey from outside of Palestine were excluded.

**Sample size calculation**: A convenience sample size in the current study was calculated using the Charan and Biswas formula (18).

**Statistical analysis**: Statistical analysis was performed using IBM SPSS statistics for windows, version 26.0 (IBM Corp, Armonk, NY, USA). A chi-square test was performed to determine the difference in categorical variables between the governorates. One-Way ANOVA test was used to determine the mean differences in quantitative variables between the two groups.

**Ethical consideration**: The study protocol was approved by the Helsinki Ethical Committee in the Gaza Strip, Palestine (Code: PHRC/HC/735/20). The participants were asked to support their participation to proceed with the online survey.

## Results

Table 1 shows the demographic characteristics by region. A total of 570 adults from the targeted regions completed the study survey; the mean age was 35.4 years (SD±9.5 years). Of those, 258 (45%), 120 (21%), and 192 (33.7%) were from the Gaza Strip, West Bank, and East Jerusalem, respectively; 321 (56.3%) were male, 249 (43.7%) were female, and 7 (3%) preferred not to mention their gender. Moreover, 432 (75.8%) participants were married while 129 (22.6%) were single, and 9 (1.6%) were divorced. On average, participants had 14.6 years (SD±5.7 years) of education and came from relatively large families (6.9 persons ±6.0 people).

**Table 1:** Characteristics of the study population by governorates

Variables		Total (n=570)	Gaza strip (n=258)	West bank (n=120)	Jerusalem (n=192)	P Value
	
		No. (%)	No. (%)	No. (%)	No. (%)	
Age (years)	Mean±SD	35.4±9.5	37.0±9.1	34.3±10.8	34.0±9.0	0.002
Gender	Male	321.0 (56.3)	228.0 (88.4)	24.0 (20.0)	69.0 (35.9)	0.001
	Female	249.0 (43.7)	30.0 (11.6)	96.0 (80.0)	123.0 (64.1)	
Marital status	Single	129.0 (22.6)	42.0 (16.3)	42.0 (35.0)	45.0 (23.4)	
	Married	432.0 (75.8)	216.0 (83.7)	72.0 (60.0)	144.0 (75.0)	0.001
	Divorced	9.0 (1.6)	0.0 (0.0)	6.0 (5.0)	3.0 (1.6)	
Years of education	Mean±SD	14.6±5.7	15.0±6.0	13.5±6.9	14.6±4.3	0.061
Profession	Unemployed	48.0 (8.4)	21.0 (8.1)	9.0 (7.5)	18.0 (9.4)	
	University student	48.0 (8.4)	18.0 (7.0)	6.0 (5.0)	24.0 (12.5)	0.120
	Officer	444.0 (77.9)	201.0 (77.9)	99.0 (82.5)	144.0 (75.0)	
	Retired	30.0 (5.3)	18.0 (7.0)	6.0 (0.5)	6.0 (3.1)	
Nature of residence area	Rural	96.0 (16.8)	39.0 (15.1)	27.0 (22.5)	30.0 (15.6)	
	Residential	462.0 (81.1)	213.0 (82.6)	87.0 (72.5)	162.0 (84.4)	0.010
	Industrial	12.0 (2.1)	6.0 (2.3)	6.0 (5.0)	0.0 (0.0)	
Type of housing	Separate apartment	366.0 (64.2)	171.0 (66.3)	63.0 (52.5)	132.0 (68.8)	
	Independent home or villa	195.0 (34.2)	87.0 (33.7)	57.0 (47.5)	51.0 (26.6)	0.001
	Converted carriage house or tent	9.0 (1.6)	0.0 (0.0)	0.0 (0.0)	9.0 (4.7)	
Family size	Mean±SD	6.9±6.0	8.8±8.2	5.4±2.6	5.2±1.9	0.001

Data are expressed as means ± SD for continuous variables and as a percentage for categorical variables. The differences between means were tested by using an independent sample t-test. The chi-square test was used to examine differences in the prevalence of different categorical variable. A P-value less than 0.05 was considered statistically significant. SD, stander deviation.

Most participants, 444 (77.9%), were professional officers, 48 (8.4%) were unemployed, 48 (8.4%) were university students, 30.0 (5.3%) were retired, and 462 (81.1%) resided in a residential area while 108 (18.9%) lived in a rural or industrial area.

With regards to the type of residence, 366 (64.2%) lived in apartments while 204 (35.6%) lived in house or villa. We noted statistically significant differences between the Gaza strip, West Bank and East Jerusalem (p < 0.05) on most socio-demographic items. (Table 1). For example, respondents from the Gaza Strip reported more years of education, came from larger families, and a higher percentage of them were male and married as compared to respondents from the West Bank and East Jerusalem.

Table 2 shows participants' attitudes toward the measures and behaviors aimed to prevent infection with COVID-19 and its consequences. The mean positive attitude for the overall sample was 94.22%; 95.24%, 95.18%, and 92.18% for the Gaza Strip, West Bank, and East Jerusalem, respectively.

**Table 2:** Attitude toward the measures and behaviors to avoid infection with Coronavirus and its consequences

Variables	Total (n=570)	Gaza strip (n=258)	West bank (n=120)	Jerusalem (n=192)	P Value

	No. (%)	No. (%)	No. (%)	No. (%)	
Physical contact with neighbors and friends should be avoided as much as possible					
Disagree	6.0 (1.1)	3.0 (1.2)	0.0 (0.0)	3.0 (1.6)	
Neither agree nor disagree	24.0 (4.2)	9.0 (3.5)	6.0 (5.0)	9.0 (4.7)	0.003
Agree	180.0 (31.6)	105.0 (40.7)	30.0 (25.0)	45.0 (23.4)	
Strongly agree	360.0 (63.2)	141.0 (54.7)	84.0 (70.0)	135.0 (70.3)	
Time must be allocated for physical activities inside the home					
Disagree	6.0 (1.1)	6.0 (2.3)	0.0 (0.0)	0.0 (0.0)	
Neither agree nor disagree	39.0 (6.8)	15.0 (5.8)	3.0 (2.5)	21.0 (10.9)	0.010
Agree	258.0 (45.3)	120.0 (46.5)	54.0 (45.0)	84.0 (43.8)	
Strongly agree	267.0 (46.8)	117.0 (45.3)	63.0 (52.5)	87.0 (45.3)	
The period of home quarantine must be used to carry out repairs that have not had time to do before the pandemic					
Disagree	6.0 (1.1)	0.0 (0.0)	0.0 (0.0)	6.0 (3.1)	
Neither agree nor disagree	42.0 (7.4)	18.0 (7.0)	9.0 (7.5)	15.0 (7.8)	0.001
Agree	207.0 (36.3)	123.0 (47.7)	27.0 (22.5)	57.0 (29.7)	
Strongly agree	315.0 (55.3)	117.0 (45.3)	84.0 (70.0)	114.0 (59.4)	
Social activities should be performed with friends and family from social networks					
Strongly disagree	3.0 (0.5)	0.0 (0.0)	0.0 (0.0)	3.0 (1.6)	
Disagree	18.0 (3.2)	3.0 (1.2)	3.0 (2.5)	12.0 (6.2)	
Neither agree nor disagree	72.0 (12.6)	33.0 (12.8)	9.0 (7.5)	30.0 (15.6)	0.001
Agree	267.0 (46.8)	141.0 (54.7)	48.0 (40.0)	78.0 (40.6)	
Strongly agree	210.0 (36.8)	81.0 (31.4)	60.0 (50.0)	69.0 (35.9)	
Healthy habits such as sleeping early and waking up early should be maintained					
Strongly disagree	6.0 (1.1)	3.0 (1.2)	0.0 (0.0)	3.0 (1.6)	
Disagree	21.0 (3.7)	3.0 (1.2)	3.0 (2.5)	15.0 (7.8)	
Neither agree nor disagree	54.0 (9.5)	21.0 (8.1)	12.0 (10.0)	21.0 (10.9)	0.001
Agree	258.0 (45.3)	138.0 (53.5)	45.0 (37.5)	75.0 (39.1)	
Strongly agree	231.0 (40.5)	93.0 (36.0)	60.0 (50.0)	78.0 (40.6)	
The habit of washing hands with soap and water should be maintained from time to time during the day					
Agree	129.0 (22.6)	69.0 (26.7)	18.0 (15.0)	42.0 (21.9)	0.038
Strongly agree	441.0 (77.4)	189.0 (73.3)	102.0 (85.0)	150.0 (78.1)	
You should avoid contact with injured or suspected patients					
Disagree	3.0 (0.5)	0.0 (0.0)	0.0 (0.0)	3.0 (1.6)	
Neither agree nor disagree	3.0 (0.5)	0.0 (0.0)	0.0 (0.0)	3.0 (1.6)	0.002
Agree	57.0 (10.0)	36.0 (14.0)	6.0 (5.0)	15.0 (7.8)	
Strongly agree	507.0 (88.9)	222.0 (86.0)	114.0 (95.0)	171.0 (89.1)	
You should avoid touching the face with your hands as much as possible					
Disagree	6.0 (1.1)	0.0 (0.0)	3.0 (2.5)	3.0 (1.6)	
Neither agree nor disagree	21.0 (3.7)	9.0 (3.5)	3.0 (2.5)	9.0 (4.7)	0.023
Agree	183.0 (32.1)	99.0 (38.4)	30.0 (25.0)	54.0 (28.1)	
Strongly agree	360.0 (63.2)	150.0 (58.1)	84.0 (70.0)	126.0 (65.6)	
Avoid being in crowded places					
Disagree	3.0 (0.5)	0.0 (0.0)	0.0 (0.0)	3.0 (1.6)	
Agree	111.0 (19.5)	72.0 (27.9)	12.0 (10.0)	27.0 (14.1)	0.001
Strongly agree	456.0 (80.0)	186.0 (72.1)	108.0 (90.0)	162.0 (84.4)	
A state of fun must be created by carrying out some recreational activities inside the house to strengthen family ties					
Neither agree nor disagree	33.0 (5.8)	18.0 (7.0)	3.0 (2.5)	12.0 (6.2)	
Agree	174.0 (30.5)	87.0 (33.7)	30.0 (25.0)	57.0 (29.7)	0.121
Strongly agree	363.0 (63.7)	153.0 (59.3)	87.0 (72.5)	123.0 (64.1)	
You should avoid getting into distress and depression					
Neither agree nor disagree	30.0 (5.3)	12.0 (4.7)	9.0 (7.5)	9.0 (4.7)	
Agree	189.0 (33.2)	87.0 (33.7)	30.0 (25.0)	72.0 (37.5)	0.192
Strongly agree	351.0 (61.6)	159.0 (61.6)	81.0 (67.5)	111.0 (57.8)	
You must maintain healthy eating and not overindulge					
Disagree	6.0 (1.1)	0.0 (0.0)	0.0 (0.0)	6.0 (3.1)	
Neither agree nor disagree	18.0 (3.2)	6.0 (2.3)	9.0 (7.5)	3.0 (1.6)	0.001
Agree	183.0 (32.1)	87.0 (33.7)	27.0 (22.5)	69.0 (35.9)	
Strongly agree	363.0 (63.7)	165.0 (64.0)	84.0 (70.0)	114.0 (59.4)	
It must comply with the instructions of the Ministry of Health and the competent authorities					
Strongly disagree	3.0 (0.5)	0.0 (0.0)	0.0 (0.0)	3.0 (1.6)	
Disagree	3.0 (0.5)	0.0 (0.0)	0.0 (0.0)	3.0 (1.6)	
Neither agree nor disagree	3.0 (0.5)	0.0 (0.0)	3.0 (2.5)	0.0 (0.0)	0.002
Agree	156.0 (27.4)	72.0 (27.9)	36.0 (30.0)	48.0 (25.0)	
Strongly agree	405.0 (71.1)	186.0 (72.1)	81.0 (67.5)	138.0 (71.9)	
Level of positive Attitude for all variables (agree and strongly agreed) by governorates %	94.22	95.24	95.18	92.18	-

Data are expressed as a percentage for categorical variables. The chi-square test was used to examine differences in the prevalence of different categorical variable. A P-value less than 0.05 was considered statistically significant.

There were significant differences between the Gaza strip, West Bank and East Jerusalem on several of the scale's items. For example, less participants from East Jerusalem agree/strongly agree that time must be allocated in the home for physical activities, social activities should be performed with friends and family through social networks, and healthy habits such as sleeping early and waking up early should continue than respondents from the Gaza Strip or West Bank.

With regards to participants' level of satisfaction with the response of the community and local authorities to combat the COVID-19 pandemic, the overall satisfaction score was 44.26%; 47.16%, 46.1%, and 39.22% for participants from the Gaza Strip, West Bank, and East Jerusalem, respectively. Overall, less than 50% of participants were satisfied with efforts aimed at allocating crews to help and care for people with disabilities, campaigns and initiatives provide psychological support for families and the attention related to the psychological support needed for families and children of crews working on the ground to fight the pandemic. We found statistically significant differences in the level of satisfaction with several measures included in the satisfaction scale between the Gaza strip, West Bank and East Jerusalem. For example, a smaller percentage of participants from the West Bank was dissatisfied or strongly dissatisfied with the implementation of initiatives and campaigns to support needy families, while a larger percentage of participants from the Gaza Strip was strongly dissatisfied or dissatisfied with campaigns and initiatives to care for the psychological support of families as well as with the care provided for older people in their homes during the pandemic. A higher percentage of West Bank participants were strongly dissatisfied with efforts to provide psychological support for families and children of crews working in the field to confront coronavirus (Table 3).

**Table 3:** Level of satisfaction with the response of the community and local authorities to combat the Corona pandemic

Variables	Total (n=570)	Gaza strip (n=258)	West bank (n=120)	Jerusalem (n=192)	P Value

	No. (%)	No. (%)	No. (%)	No. (%)	
Implementation of initiatives and campaigns to support needy families					
Very dissatisfied	48.0 (8.4)	30.0 (11.6)	6.0 (5.0)	12.0 (6.2)	
Dissatisfied	87.0 (15.3)	33.0 (12.8)	15.0 (12.5)	39.0 (20.3)	
Unsure	147.0 (25.8)	48.0 (18.6)	42.0 (35.0)	57.0 (29.7)	0.001
Satisfied	204.0 (35.8)	111.0 (43.0)	36.0 (30.0)	57.0 (29.7)	
Very satisfied	84.0 (14.7)	36.0 (14.0)	21.0 (17.5)	27.0 (14.1)	
Allocation of crews to help and care for people with disabilities					
Very dissatisfied	42.0 (7.4)	21.0 (8.1)	6.0 (5.0)	15.0 (7.8)	
Dissatisfied	93.0 (16.3)	45.0 (17.4)	21.0 (17.5)	27.0 (14.1)	
Unsure	174.0 (30.5)	66.0 (25.6)	39.0 (32.5)	69.0 (35.9)	0.447
Satisfied	183.0 (32.1)	90.0 (34.9)	39.0 (32.5)	54.0 (28.1)	
Very satisfied	78.0 (13.7)	36.0 (14.0)	15.0 (12.5)	27.0 (14.1)	
Carry out campaigns and initiatives to care for the psychological support of families					
Very dissatisfied	51.0 (8.9)	24.0 (9.3)	12.0 (10.0)	15.0 (7.8)	
Dissatisfied	84.0 (14.7)	27.0 (10.5)	21.0 (17.5)	36.0 (18.8)	
Unsure	189.0 (33.2)	90.0 (34.9)	27.0 (22.5)	72.0 (37.5)	0.009
Satisfied	171.0 (30.0)	78.0 (30.2)	48.0 (40.0)	45.0 (23.4)	
Very satisfied	75.0 (13.2)	39.0 (15.1)	12.0 (10.0)	24.0 (12.5)	
Allocation of green areas for Hiking and physical activities					
Very dissatisfied	78.0 (13.7)	30.0 (11.6)	24.0 (20.0)	24.0 (12.5)	
Dissatisfied	189.0 (33.2)	84.0 (32.6)	33.0 (27.5)	72.0 (37.5)	
Unsure	153.0 (26.8)	69.0 (26.7)	33.0 (27.5)	51.0 (26.6)	0.236
Satisfied	78.0 (13.7)	42.0 (16.3)	12.0 (10.0)	24.0 (12.5)	
Very satisfied	72.0 (12.6)	33.0 (12.8)	18.0 (15.0)	21.0 (10.9)	
Paying interest in continuing students' scientific attainment through the implementation of the distance learning plan					
Very dissatisfied	45.0 (7.9)	18.0 (7.0)	12.0 (10.0)	15.0 (7.8)	
Dissatisfied	102.0 (17.9)	51.0 (19.8)	15.0 (12.5)	36.0 (18.8)	
Unsure	135.0 (23.7)	54.0 (20.9)	33.0 (27.5)	48.0 (25.0)	0.235
Satisfied	192.0 (33.7)	96.0 (37.2)	33.0 (27.5)	63.0 (32.8)	
Very satisfied	96.0 (16.8)	39.0 (15.1)	27.0 (22.5)	30.0 (15.6)	
Paying attention to the psychological support of families and children of crews working in the field to confront Coronavirus					
Very dissatisfied	69.0 (12.1)	30.0 (11.6)	21.0 (17.5)	18.0 (9.4)	
Dissatisfied	108.0 (18.9)	48.0 (18.6)	24.0 (20.0)	36.0 (18.8)	
Unsure	168.0 (29.5)	66.0 (25.6)	36.0 (30.0)	66.0 (34.4)	0.006
Satisfied	123.0 (21.6)	72.0 (27.9)	12.0 (10.0)	39.0 (20.3)	
Very satisfied	102.0 (17.9)	42.0 (16.3)	27.0 (22.5)	33.0 (17.2)	
The community complies with the instructions of the Ministry of Health and the authorities concerned with staying at home					
Very dissatisfied	75.0 (13.2)	36.0 (14.0)	12.0 (10.0)	27.0 (14.1)	
Dissatisfied	123.0 (21.6)	54.0 (20.9)	21.0 (17.5)	48.0 (25.0)	
Unsure	135.0 (23.7)	57.0 (22.1)	30.0 (25.0)	48.0 (25.0)	0.061
Satisfied	150.0 (26.3)	81.0 (31.4)	33.0 (27.5)	36.0 (18.8)	
Very satisfied	87.0 (15.3)	30.0 (11.6)	24.0 (20.0)	33.0 (17.2)	
Carrying out campaigns and initiatives to clean and sterilize streets and public places					
Very dissatisfied	36.0 (6.3)	12.0 (4.7)	0.0 (0.0)	24.0 (12.5)	
Dissatisfied	78.0 (13.7)	36.0 (14.0)	9.0 (7.5)	33.0 (17.2)	
Unsure	126.0 (22.1)	54.0 (20.9)	30.0 (25.0)	42.0 (21.9)	0.001
Satisfied	210.0 (36.8)	114.0 (44.2)	48.0 (40.0)	48.0 (25.0)	
Very satisfied	120.0 (21.1)	42.0 (16.3)	33.0 (27.5)	45.0 (23.4)	
Implementing special procedures for the care of older persons living in the homes of the elderly					
Very dissatisfied	57.0 (10.0)	21.0 (8.1)	15.0 (12.5)	21.0 (10.9)	
Dissatisfied	87.0 (15.3)	51.0 (19.8)	12.0 (10.0)	24.0 (12.5)	
Unsure	180.0 (31.6)	72.0 (27.9)	33.0 (27.5)	75.0 (39.1)	0.011
Satisfied	126.0 (22.1)	63.0 (24.4)	33.0 (27.5)	30.0 (15.6)	
Very satisfied	120.0 (21.1)	51.0 (19.8)	27.0 (22.5)	42.0 (21.9)	
Level of satisfaction for all variables (satisfied and very satisfied) by governorates %	44.26	47.16	46.1	39.22	-

Data are expressed as a percentage for categorical variables. The chi-square test was used to examine differences in the prevalence of different categorical variable. A P-value less than 0.05 was considered statistically significant.

## Discussion

To the best of our knowledge, the current study is one of the first addressing attitudes toward and satisfaction with COVID-19 mitigation measures in Palestine. The Palestinian ministry of health (MOH) announced the first COVID cases discovered in Palestine in March 2020. Since then, the number of cases has increased with over 335176 confirmed cases and over 3748 deaths as of May. 25, 2021 (19). Overall, we found a relatively positive attitude toward the measures with slight variation across the three study areas.

The majority of respondents agreed with most of the preventive measures in reducing the chances of being infected and had a positive attitude toward the protective measures requested by local health authorities. These findings are consistent with previous studies in Egypt (20) and India (21) that documented a positive attitude toward most COVID-19 preventive measures.

In our study, most respondents agreed that avoiding crowded places is essential for preventing the spread of COVID-19. This finding is supported by another survey by Hager et al. (2020), which found the majority of the respondents practiced self-isolation and social distancing (22).

In addition, most respondents in our study agreed with the need to avoid contact with people with suspected or confirmed COVID-19 diagnosis and avoid touching their face with hands. Furthermore, a large percentage of respondents agreed that they must comply with the instructions of the MOH and the authorities. These findings are consistent with those reported by Hager et al. (2020) based on a study with a bi-national survey in Africa (22). The authors of the current study believe that the positive attitude observed in this study may be attributed to the relatively high education level of the respondents.

Although this study was conducted during the compulsory lockdown in Palestine, the positive attitude of the Palestinians could be seen in a positive attitude level toward most of the preventive measures.

The findings of the present study show that the level of satisfaction with the response of the community and local authorities to combat the COVID-19 pandemic was 44.26%, distributed as 47.16%, 46.1%, and 39.22% in the Gaza Strip, West Bank, and East Jerusalem, respectively. These results show a similar satisfaction score for the Gaza Strip and the West Bank, while the level of satisfaction was less in East Jerusalem. This is somewhat in line with the findings of another study by Izhar et al. (2020), which found that only 19.5% were satisfied with the social distancing measures in Pakistan (23). Less than one-third (31%) were satisfied with the PPE available to them. In addition, our findings are supported by another study by Hager et al. (2020), which showed that 22% of the respondents were satisfied with their country's handling of the pandemic (22).

In our study, most participants were unsatisfied or unsure about the support provided for people with disabilities, psychological support for families, and the psychological support offered to families and children of crews working on the ground to fight the pandemic.

Our study has some limitations that should be considered. The study was conducted online, and it was not possible to verify the responses. In addition, the study was advertised on social media platforms, and the educational level of participants is higher than that for the general population. Therefore, our sample might be more representative of educated people with access to the internet and social media. Having said that, our study has several strengths, including covering a wide geographic area representing Palestinians in the Gaza Strip, West Bank and East Jerusalem. In addition, the study is one of the first to shed light on attitudes toward and satisfaction with COVID-19 mitigation efforts in the area.

The current study demonstrated suboptimal levels of satisfaction toward the taken procedures to tackle COVID-19 in Palestine. Our results constitute a call for action by the local authorities and community organizations to design interventions and programs to address the needs of people with disabilities and provide psychological support for residents, especially for families of those in the front lines. Providing psychological support and psychoeducation campaigns addressing the detrimental impact of the COVID-19 pandemic on mental health may reduce the psychological symptoms and stigma associated with the pandemic.
